# Correction: A vertical WSe_2_–MoSe_2_ p–n heterostructure with tunable gate rectification

**DOI:** 10.1039/c8ra90067a

**Published:** 2018-08-13

**Authors:** Hailiang Liu, Sajjad Hussain, Asif Ali, Bilal Abbas Naqvi, Dhanasekaran Vikraman, Woonyoung Jeong, Wooseok Song, Ki-Seok An, Jongwan Jung

**Affiliations:** Graphene Research Institute, Sejong University Seoul 143-747 Republic of Korea jwjung@sejong.ac.kr; Institute of Nano and Advanced Materials Engineering, Sejong University Seoul 143-747 Republic of Korea; Division of Electronics and Electrical Engineering, Dongguk University-Seoul Seoul 04620 Republic of Korea; Thin Film Materials Research Center, Korea Research Institute of Chemical Technology Daejon 305-600 Korea

## Abstract

Correction for ‘A vertical WSe_2_–MoSe_2_ p–n heterostructure with tunable gate rectification’ by Hailing Liu *et al.*, *RSC Adv.*, 2018, **8**, 25514–25518.

The authors regret that Hailiang Liu’s name was spelled incorrectly in the original article; the corrected version is shown above.

The Royal Society of Chemistry apologises for these errors and any consequent inconvenience to authors and readers.

## Supplementary Material

